# How superdiffusion gets arrested: ecological encounters explain shift from Lévy to Brownian movement

**DOI:** 10.1098/rspb.2013.2605

**Published:** 2014-01-07

**Authors:** Monique de Jager, Frederic Bartumeus, Andrea Kölzsch, Franz J. Weissing, Geerten M. Hengeveld, Bart A. Nolet, Peter M. J. Herman, Johan van de Koppel

**Affiliations:** 1 Spatial Ecology Department, Royal Netherlands Institute for Sea Research (NIOZ), PO Box 140, 4400 AC Yerseke, The Netherlands; 2 Theoretical Biology Group, University of Groningen, Nijenborgh 7, 9747 AG Groningen, The Netherlands; 3 Community and Conservation Ecology Group, Centre for Ecological and Evolutionary Studies, University of Groningen, Nijenborgh 7, 9747 AG Groningen, The Netherlands; 4 Institute of Integrative Biology, ETH Zürich, Universitaetstrasse 16, 8092 Zürich, Switzerland; 5 Center for Advanced Studies of Blanes (CEAB-CSIC), Accés Cala Sant Francesc, 14, 17300 Blanes, Girona, Spain; 6 Department of Animal Ecology, Netherlands Institute of Ecology (NIOO-KNAW), PO Box 50, 6700 AB Wageningen, The Netherlands; 7 Project Group Movement Ecology, Netherlands Institute of Ecology (NIOO-KNAW), PO Box 50, 6700 AB Wageningen, The Netherlands

**Keywords:** Brownian motion, Lévy walk, animal movement, *Mytilus edulis*, search efficiency, resource density

## Abstract

Ecological theory uses Brownian motion as a default template for describing ecological movement, despite limited mechanistic underpinning. The generality of Brownian motion has recently been challenged by empirical studies that highlight alternative movement patterns of animals, especially when foraging in resource-poor environments. Yet, empirical studies reveal animals moving in a Brownian fashion when resources are abundant. We demonstrate that Einstein's original theory of collision-induced Brownian motion in physics provides a parsimonious, mechanistic explanation for these observations. Here, Brownian motion results from frequent encounters between organisms in dense environments. In density-controlled experiments, movement patterns of mussels shifted from Lévy towards Brownian motion with increasing density. When the analysis was restricted to moves not truncated by encounters, this shift did not occur. Using a theoretical argument, we explain that any movement pattern approximates Brownian motion at high-resource densities, provided that movement is interrupted upon encounters. Hence, the observed shift to Brownian motion does not indicate a density-dependent change in movement strategy but rather results from frequent collisions. Our results emphasize the need for a more mechanistic use of Brownian motion in ecology, highlighting that especially in rich environments, Brownian motion emerges from ecological interactions, rather than being a default movement pattern.

## 1. Introduction

Traditionally, ecologists apply Brownian motion and diffusive dispersal as default models for animal movement [1,2], both at individual and at population levels [3–5]. Recently, however, empirical studies have shown that animal movement can strongly deviate from Brownian motion [6], revealing superdiffusive, Lévy-like movement in resource-poor environments, but standard Brownian motion when resource availability is high [7–11]. Animal ecologists have explained this change from Lévy to Brownian motion by an active shift in individual movement strategy, reflecting the assumption that different movement strategies are optimal under different environmental conditions [10–13]. In heterogeneous, resource-poor environments, Lévy movement will typically be more efficient than a Brownian walk because it provides faster dispersal and prevents revisiting the same sites [14]. In resource-rich environments, a Brownian walk may be equally or even more efficient than a Lévy walk, because large steps (which are the hallmark of Lévy movement) provide little benefit under these circumstances [11].

Physical theory offers an alternative, more parsimonious explanation for the occurrence of Brownian motion in resource-rich environments. Einstein, followed by Langevin, theorized that Brownian motion in solutes results from collisions between particles [15,16]. Likewise, Brownian motion in ecology might result from frequent ‘collisions’ of animals with the resources they are searching for (food, shelter or conspecifics), or with items that they are trying to avoid (e.g. territory boundaries [17]). Untangling whether the observed movement patterns in searching animals reflect adaptation of intrinsic movement strategies, or are the consequence of changing encounter (collision) rates with resources, is crucial both for sound mechanistic understanding of Brownian motion and for predicting animal movement patterns in ecosystems where resource availability varies in space or time.

Here, we provide evidence that, as in physics, Brownian walks in animal movements can be caused by frequent encounters, rather than being the result of adaptation to high-density conditions. In density-controlled experiments with young mussels (*Mytilus edulis*), we were able to distinguish between intrinsic movement strategy and the effects of resource density by separating the movement steps that were truncated by encounters from those that were terminated spontaneously. Recently, it was shown that the individual movement of young mussels can be approximated by a simple Lévy walk [18] (or a more complex multi-scale walk, which provides an even better fit [19,20]). The movement of individual mussels results in a self-organized mussel bed with a regular labyrinth-like pattern where local aggregation yields protection against wave stress and predation while it reduces competition for algal food resources [21–23]. As the movement of individual mussels can be experimentally studied in considerable detail, this system provides a unique opportunity to investigate how animal movement patterns are affected by truncation of moves owing to encounters.

This paper is structured as follows. First, we describe movement of young mussels observed in density-controlled experiments, revealing that movement patterns are affected by changes in the density of mussels. By distinguishing between obstructed and unobstructed movement steps, we investigate the relationship between intended and realized movement patterns. Second, we create an individual-based model of self-organized pattern formation in mussel beds to examine whether mussel density could cause a change in the efficiency of Brownian and Lévy walks, explaining a possible active shift in mussel movement strategy. Third, we use a general argument to demonstrate that the interplay between *any* intrinsic movement strategy and frequent ecological encounters will often result in Brownian motion.

## 2. Experiments

### (a) Methods

Using mesocosm experiments, we investigated how mussel movement patterns are affected by mussel density. Young blue mussels (*M. edulis*) of approximately 1.5 cm in length were obtained from wooden wave-breaker poles on the beaches near Vlissingen, The Netherlands (51°46′ N, 3°53′ E). After careful separation and cleaning, the mussels were kept in containers and fed live cultures of diatoms (*Phaeodactylum tricornutum*) daily. Fresh, unfiltered seawater was supplied to the container at a rate of approximately 1 l min^−1^; a constant water temperature of 16°C was maintained during the experiments. At the start of each experiment, mussels were spread homogeneously over an 80 × 60 cm red PVC sheet in a 120 × 80 × 30 cm container. We used a red PVC sheet to provide a contrast-rich surface for later analysis and considered only the movements of the mussels within this 80 × 60 cm arena. The container was illuminated using fluorescent lamps. Mussel movement was recorded by photographing the mussels at 1 min intervals for a duration of 300 min; we used a Logitech QuickCam 9000 Pro webcam (www.logitech.com), which was positioned about 60 cm above the water surface.

We derived the step lengths by calculating the distance between two reorientation events (e.g. where a mussel clearly changes its direction of movement) using Turchin's angle method [18,24]. First, the observed movement path is discretized into steps on basis of changes in the angle (*α*) of the movement path at observed position *i* using the prior (*i* − 1) and the subsequent (*i* + 1) observed locations as follows:2.1
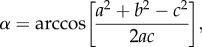
where *a* is the length between position *i* and *i* + 1, *b* is the length between position *i* − 1 and *i* + 1 and *c* is the length between positions *i* − 1 and *i*. Whenever *α* was larger than a threshold angle *α*_T_, a new step is considered to start. Following Turchin's approach [24], we used *α*_T_ = *π*/5 for our step length calculations, as this value minimized autocorrelation between subsequent turns. Using other threshold angles did not change our conclusions.

We studied the changes in the statistical properties of the observed movement pattern by recording 10 individual movement trajectories for five different density treatments each (0, 1.3, 2.0, 3.3 and 5.2 kg m^−2^, approx. 1, 950, 1550, 2500 and 3850 mussels m^−2^) during the initial 300 min of pattern formation [23]. When a mussel encountered an obstacle, for example a conspecific, it was forced to truncate its step, which will probably alter the properties of the movement pattern. We used the complementary cumulative distribution function (CCDF) of the observed step lengths of each individual mussel in the five density treatments to illustrate the observed movement patterns. This CCDF is a preferred method for fitting power distributions as it provides a more reliable representation of movement patterns than other portraying methods [3]. For each step length *l*, the CCDF(*l*) of the observed step lengths in each density treatment indicates the fraction of step lengths that were at least as long as *l*. Using maximum-likelihood methods, we estimated the scaling exponent *μ* of a power-law step length distribution,2.2
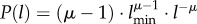
where *l* is the step length and *l*_min_ is the minimal step length of young mussels (*l*_min_ ≤ *l*) [3,18,25,26]. The step length distribution corresponds to a Lévy walk for *1* < *µ* < 3 and it approximates a Brownian walk when *μ* > 3 [27]. We apply a simple power-law model rather than a more complex composite model because we are interested in the change of general statistical properties with mussel density rather than in a detailed statistical description of mussel movement [18–20]. First, we kept the minimal step length constant at the fixed value *l*_min_ = 3 mm. Given *l*_min_, the exponent *μ* can be estimated from the likelihood function [25,26,28,29]2.3
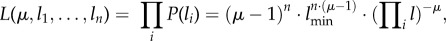
where 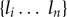 are the observed step lengths. Taking the natural logarithm of *L* and maximizing with respect to *µ* yields the maximum-likelihood estimate2.4
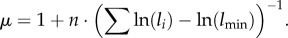
To check for the robustness of our results, we also fitted the observed step length distribution to a power law where the value of *l*_min_ was estimated separately for each individual trajectory (by equating *l*_min_ with the minimal observed step length). Our conclusions were not affected in any way.

By labelling steps as truncated whenever the step ended directly in front of another mussel, we were able to distinguish pure, non-truncated steps from those truncated by collisions with conspecifics. For the same 10 individuals in the five density treatments (50 mussels in total), we split the steps into truncated and non-truncated steps, examining the distributions separately.

### (b) Results

**Figure 1.**
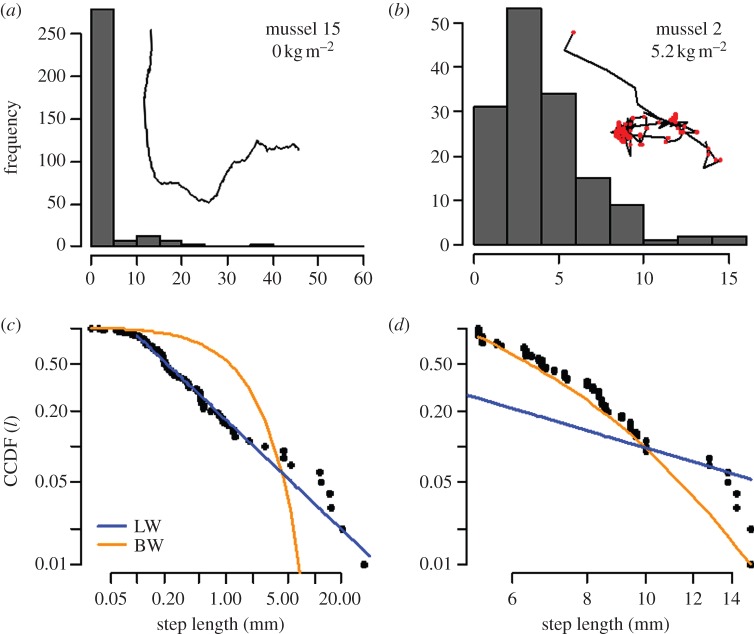
Step length distributions and model fits for movement trajectories at two mussel densities. Step length frequency distributions of mussel 15 in the 0 kg m^−2^ treatment (*a*) and mussel 2 in the 5.2 kg m^−2^ treatment (*b*), together with an illustration of the movement paths (red dots indicate encounters with conspecifics). The fitted lines to the CCDFs of the step lengths of mussel 15 (*c*) and mussel 2 (*d*) indicate how well the movement trajectories are represented by a Lévy walk (LW) and a Brownian walk (BW).

**Figure 2.**
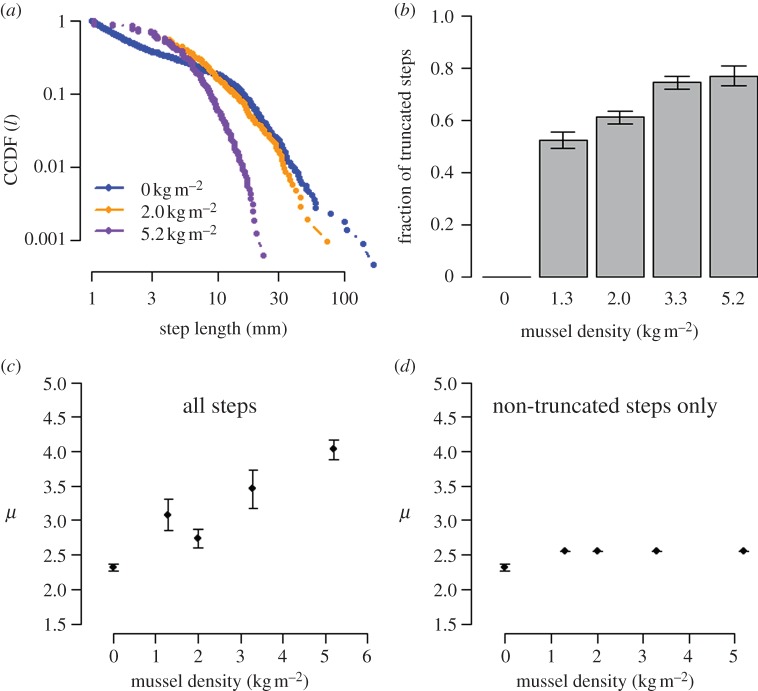
Effect of mussel density on individual movement trajectories. (*a*) CCDF of the pooled step lengths of moving mussels measured for three density treatments. With increasing mussel density, the fraction of long steps decreases. (*b*) Estimated scaling exponent *μ* as a function of mussel density; *μ* increases with mussel density (linear regression, *β*_1_ = 0.73, *r* = 0.46, d.f. = 46, *p* < 0.001; bars indicate average *μ* per density group ±s.e.) and takes on values beyond 3 at high densities. (*c*) The fraction of steps that are truncated by collisions increases with mussel density (bars indicate means ± s.e.). (*d*) When considering the non-truncated steps only, the scaling exponent *μ* remains approximately constant (linear regression, *β*_1_ = 0.18, *r* = 0, d.f. = 26, *p* = 0.593; bars indicate average *μ* per density group ±s.e.).

Our mesocosm experiments illustrate that the observed movement patterns are strongly affected by mussel density (figures 1 and 2; see the electronic supplementary material, table S1). Long steps occur less frequently with increasing mussel density (figure 2*a*). The scaling exponent *μ* increases with mussel density from a value below 2.5 at low densities to values above 3.5 at high densities (figure 2*b*). As a second test of our hypothesis that observed movement trajectories become more Brownian-like with increased resource density, we used the Akaike information criterion for deciding whether the individual trajectories in each density class were better fitted by a power law or by an exponential distribution (corresponding to a Brownian walk). In 83% of the movement trajectories in the lowest density treatment, a Lévy walk provided a better fit to the step length data than a Brownian walk. By contrast, 75% of the tracks in the high-density treatment were better approximated by a Brownian walk than by a Lévy walk. Again, we conclude that movement trajectories become more Brownian-like with increasing mussel density.

Closer examination of the movement data indicates that the change of step length distribution with mussel density results from the frequent truncation of step lengths at high densities (figure 2*c*,*d*). The fraction of truncated steps increases with mussel density (figure 2*c*), presumably because the number of encounters leading to an interruption of the movement increases with density. When only considering non-truncated steps, mussel movement does not significantly differ between density treatments (figure 2*d*). We conclude that the intrinsic movement strategy of the mussels does not change with density and that the observed change from Lévy-like to Brownian-like movement results solely from the increased mussel encounter rates at high density.

## 3. A model of mussel movement

### (a) Methods

Using a well-established model for mussel movement [18], we investigated whether an active switch from Lévy to Brownian movement at high densities is more efficient than the persistent use of Lévy movement. We ran individual-based computer simulations for a range of values of the scaling exponent *μ* and at various densities, where we repeated each simulation 10 times to account for stochasticity. Whenever a displacement was restricted by the presence of a conspecific, the step was truncated. In each simulation, we determined the sum *D* of all displacements required before the mussels settled in a stable pattern. The inverse of *D* can be viewed as a measure of the patterning efficiency of the movement strategy under consideration [18,30].

### (b) Results

**Figure 3.**
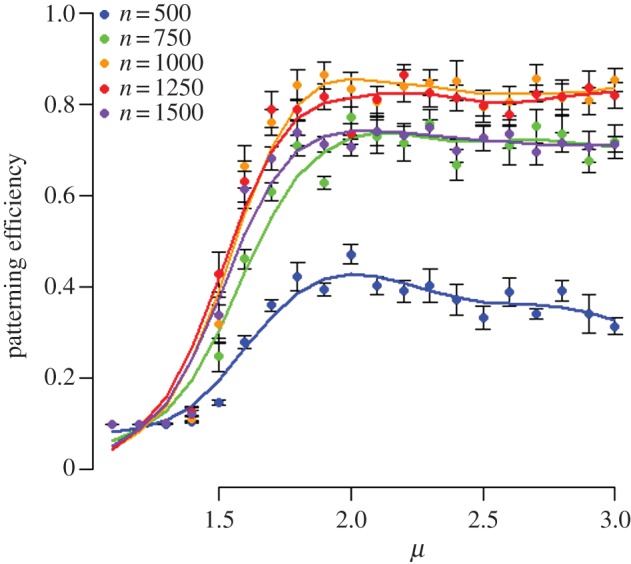
Patterning efficiency as a function of the scaling exponent *μ* in model simulations for five different mussel densities. At low mussel density (*n* = 500), a LW with *μ* ≈ 2 has the highest patterning efficiency, i.e. this movement strategy creates a spatial pattern with a minimum of displacements. At higher densities, a LW with *μ* ≈ 2 still appears optimal, but most other movement strategies (including a BW) perform equally well. Bars indicate means of 10 simulations ± s.d.; lines illustrate cubic smoothing splines through the model results. Patterning efficiency, measured as the inverse of the distance *D* moved per mussel until a pattern was formed, was normalized by dividing by the largest efficiency found in all simulations.

Brownian movement is often assumed to be more efficient in dense environments; some researchers thus argue that animals switch from Lévy to Brownian movement when encountering areas of higher resource density. However, simulations with our individual-based model [18] of mussel movement demonstrate that Lévy movement is at least as efficient as Brownian motion at all densities. At low densities, a Lévy walk with exponent *μ ≈ 2* is the most efficient movement strategy (figure 3). At higher densities, all movement strategies with 2 ≤ *μ* ≤ 3 lead to Brownian-like movement patterns and therefore have a similar patterning efficiency; hence, the simulations do not support the hypothesis that Brownian movement strategies lead to more efficient aggregation than Lévy movement strategies. This implies that there is no necessity to switch to a Brownian strategy with increasing density and the mussels in our experiments do not behave suboptimally when using a Lévy walk at high densities (figure 2*d*).

## 4. A general argument

By means of a general argument, it can be seen that the transition from non-Brownian to Brownian motion at high densities is a general phenomenon and not restricted to mussel movement. Consider a population of animals where the individuals have a certain intrinsic movement strategy, for example a Lévy walk with a given exponent *μ*. If all individuals could complete their movement steps uninterrupted, this movement strategy would result in a step length distribution with a complementary cumulative distribution function CCDF_intended_(*l*) (as in figure 2*a*, CCDF(*l*) corresponds to the probability that a step is longer than or equal to *l*). Suppose now that an animal terminates its movement whenever it encounters its desired target, such as food or shelter. (The same arguments apply when moves are terminated owing to encounters with obstacles or the presence of a potential danger, such as a predator or a rival.) If the encounters of the moving animals with the target objects are random, the probability that an intended step of length *l* will *not* be terminated is given by the zero term of a Poisson distribution: e*^−kAl^*, where *A* is the density of target objects and *k* is a constant of proportionality that reflects aspects such as the search window of the animal or the size and visibility of the target objects. As a consequence, the CCDF of the realized (and observed) step length distribution is given by4.5
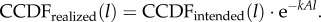


**Figure 4.**
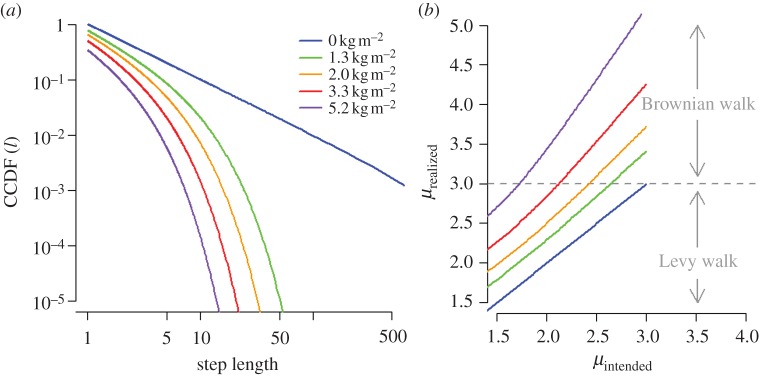
Difference between intended and realized step length distribution for various densities of the target object. (*a*) CCDFs of the realized step lengths of organisms using a LW with scaling exponent *μ*_intended_ = 2 as their intrinsic movement strategy. Only at zero density, the realized CCDF corresponds to the intended CCDF, while the fatness of the tail of the distribution strongly decreases at higher densities. The realized CCDF approximately correspond to the CCDF of a power law with scaling exponent *μ*_realized_ = 2.5, 2.9, 3.0 and 3.5 for the increasing densities, respectively. (*b*) Relationship between intrinsic scaling exponent *μ*_intended_ and realized scaling exponent *μ*_realized_ for various object densities. Movement patterns are often classified as a LW when the estimated value of *μ* is between 1 and 3 and as BW when *μ* > 3.

As step lengths will become shorter owing to the termination of steps by encounters, the realized step length distribution will have a different signature than the intended step length distribution. In particular, intended longer steps will be terminated more often than intended shorter steps, and the probability that a step is terminated will depend on the density of target objects. For large densities of the target object, the exponential term becomes dominant and forces the tail of the CCDF towards the exponential distribution that is characteristic of Brownian walks (figure 4). For example, the CCDF of an intended Lévy walk with exponent *μ*_intended_ = 2 results in a realized CCDF that, owing to the termination of steps by encounters with the target object, resembles the CCDF of a Lévy walk with a larger exponent *μ*_realized_ (figure 4*a*). In more general terms, an intended movement strategy that is not Brownian at all takes on the signature of Brownian motion when intended movement steps are frequently terminated because of a high density of target objects (figure 4*b*).

## 5. Discussion

Einstein demonstrated that Brownian motion of dissolved particles can be explained by heat-driven collisions of these particles with the molecules of the liquid [15,16]. Despite obvious differences between movement in particles and organisms, our study shows that in analogy to physics, encounters between organisms result in Brownian motion, in particular when found in encounter-rich environments. We observed that under controlled, experimental conditions, mussel movement patterns shifted from Lévy to Brownian motion with increasing mussel density. By separating truncated from non-truncated steps, we were able to show that this change in movement pattern is entirely the consequence of increased encounter rate, as we did not observe a shift in intrinsic movement strategy. We furthermore demonstrated the universality of this principle with a simple argument, showing that in general, encounters lead to Brownian motion in animal movement patterns.

The shift from Lévy-like to Brownian movement with increasing density has so far been explained as an adaptation to increased resource availability. Animals are considered to adapt to increased encounters with food items by refraining from large-scale movement steps, hence leading to adaptive Brownian walks [12,31]. However, our study provides a different perspective on the observed shift from Lévy-like to Brownian movement. When encounter rates are low, the observed movement pattern reflects the intrinsic search strategy, which can strongly deviate from Brownian movement. When encounter rates are high, the signature of the intrinsic search strategy is lost; large movement steps are frequently truncated by encounters and the movement pattern resembles Brownian motion irrespective of the underlying intrinsic strategy. This has important implications for ecological theory, as here Brownian motion is not a default, intrinsic movement mode that underlies animal dispersal but emerges from ecological encounters between organisms, such as encounters with food items or interference with conspecifics, similar to the physical obstruction of mussel movement observed in our study.

The explanation of encounters driving Brownian motion can clarify observations from a number of terrestrial and marine studies. For instance, studies by Bartumeus *et al.* [8], De Knegt *et al.* [9] and Humphries *et al.* [10–11] illustrate that microzooplankton, goats, marine predators and albatrosses all exhibit Brownian motion in areas with high food density and Lévy-like movement in resource-poor environments. These studies highlight that an increased prevalence of Brownian motion in resource-rich environments is a general trend in ecological systems. Our explanation that encounters obscure innate movement strategy into an observed movement pattern that closely resembles a Brownian walk rationalizes this universal trend. As a variety of ecological encounters, such as predator–prey interactions, mating or aggregation, are prone to occur in real ecosystems, observed animal movement patterns will always deviate from the employed intrinsic movement strategy. Especially in rich environments, resource encounters may alter the movement pattern extensively. Hence, our study not only illustrates the generality of this principle, but also highlights the importance of ecological interactions in shaping movement patterns of organisms throughout nature.

While density-dependence of demographic processes, such as growth and predation, forms the cornerstone of ecological theory, animal movement and dispersal are typically approximated by density-independent linear diffusion, based on the assumption of Brownian motion. This study, in combination with previous work [7–11,18,23] shows that for many organisms, this assumption is not valid; both movement rates and movement characteristics may change as a function of the local density of food items or conspecifics, being either through ecological encounters as advocated in this paper, or through adaptation of movement [10]. As a consequence, movement characteristics at the population level may change with density, for instance from superdiffusive dispersal at low encounter rates, to more conservative linear diffusion at high encounter rates. This can have important consequences for, for instance, the rate of spread of infectious diseases and invasive species or the formation of self-organized patterns. As the underlying movement strategy will often be masked under high-density conditions and organisms thus might behave differently under low-density conditions, one must be careful not to draw too far-reaching conclusions from movement patterns observed in dense environments. A more mechanistic understanding of ecological movement, facilitated by current improvements in techniques to monitor moving animals, will greatly expand our ability to examine, model and comprehend animal movement patterns and their influence on other ecological processes.
